# Moonlighting

**Published:** 1881-08

**Authors:** 


### "MOONLIGHTING."

MOONLIGHTING is a peculiar industry that owes its existence to the patent laws. The late Colonel E. A. Roberts introduced the use of nitro-glycerine torpedoes in increasing the yield of oil wells. When the great flowing wells of Oil Creek, after draining the petroleum pools of the lower field for three years, had exhausted the supply, as was supposed, Colonel Roberts experimented on an abandoned well with a quantity of nitro-glycerine, confined in a tin shell and exploded by concussion. The explosion was followed by a flow of oil, and the old well yielded thirty barrels a day for several years afterward. The nitro-glycerine had shattered the oil-bearing rock and opened the paraffine-clogged veins. While serving in the army, Colonel Roberts noticed that a bombshell exploding beneath water invariably spent its force on the bottom of the stream, throwing up mud and stones in great quantities. This was due, he supposed, to the solid fluid tamping above the explosive. It was this idea that led him to try the experiment of nitro-glycerine at the bottom of oil wells, beneath hundreds of feet of fluid tamping—oil and water collected in the well. He obtained patents on his device. The validity of the patents was questioned, and nitro-glycerine torpedoes were used by others without paying royalty to Roberts. He brought nearly 5,000 suits to protect his rights. One of these, as a test, was carried through all the State courts and to the United States Supreme Court. Roberts won in every court, and nearly a million dollars in royalties was recovered.

The monopoly in nitro-glycerine torpedoes led to the illicit use of them in wells. Men without fear of death or regard for law went into the business of "shooting" wells for producers who did not care to pay tribute to Roberts. Any one has a right to manufacture nitro-glycerine and to place torpedoes in wells. In the exploding of them lies the liability to prosecution and penalty. The moonlighter is always ready to contract for the shooting of a well. He carries his nitro-glycerine in wagons made especially for the purpose. They are buckboards, with cushioned apartments under the seat, in which the cans are placed. The roads of the oil regions would scarcely be called roads elsewhere. When not hub deep with mud, they are stretches of deep ruts and gulleys and projecting rocks. Drawn by powerful horses, these wagons, loaded with sixty or a hundred quarts of one of the most destructive explosives known, and which a sudden jar is at any moment likely to explode, are driven by their reckless owners over these roads in the darkest nights at the top of their horses' speed. The men work at night always. They are called moon-lighters, but the absence of the moon does not prevent them from undertaking a job.

